# Vitamin B6 deficiency produces metabolic alterations in *Drosophila*

**DOI:** 10.1007/s11306-025-02236-0

**Published:** 2025-03-23

**Authors:** Giulia Tesoriere, Eleonora Pilesi, Michele De Rosa, Ottavia Giampaoli, Adriano Patriarca, Mariangela Spagnoli, Federica Chiocciolini, Angela Tramonti, Roberto Contestabile, Fabio Sciubba, Fiammetta Vernì

**Affiliations:** 1 Department of Biology and Biotechnology “Charles Darwin”, Sapienza University of Rome, 00185 Rome, Italy; 2 Department of Chemistry, Sapienza University of Rome, Piazzale Aldo Moro 5, 00185 Rome, Italy; 3 NMR-Based Metabolomics Laboratory (NMLab), Sapienza University of Rome, Rome, Italy; 4 Department of Environmental Biology, Sapienza University of Rome, Piazzale Aldo Moro 5, 00185 Rome, Italy; 5 Department of Occupational and Environmental Medicine, Epidemiology and Hygiene, INAIL, Via Fontana Candida 1, 00078 Monte Porzio Catone, Italy; 6 Institute of Molecular Biology and Pathology, Consiglio Nazionale delle Ricerche, 00185 Rome, Italy; 7 Department of Biochemical Sciences “A. Rossi Fanelli”, Sapienza, University of Rome, 00185 Rome, Italy

**Keywords:** Vitamin B6, Pyridoxal 5’-phosphate (PLP), 4-deoxypyridoxine (4DP), Metabolomic analysis, NMR spectroscopy, *Drosophila melanogaster*

## Abstract

**Introduction:**

Pyridoxal 5’-phosphate (PLP), the biologically active form of vitamin B6 is involved in 4% of cellular enzymatic activities and its deficiency is responsible for or contributes to several human diseases. The study of underlying mechanisms is still in its infancy and requires suitable model organisms. In *Drosophila* the deficiency of vitamin B6 produces chromosome aberrations and hallmarks of human diseases including diabetes and cancer. However, the effects of vitamin B6 deficiency have never been examined at a metabolic level.

**Objectives:**

This study evaluates the metabolic changes in vitamin B6 deficient *Drosophila* larvae with the aim of validating flies as a suitable model for diseases associated to vitamin B6 deficiency.

**Methods:**

To induce vitamin B6 deficiency we fed *Drosophila* wild type larvae with 4-deoxypyridoxine (4DP), a PLP antagonist. By HPLC analysis we verified that the 4DP treatment was effective in inducing vitamin B6 deficiency. Using an NMR-based metabolomic approach we compared the metabolites in larval extracts from untreated and 4DP-fed larvae.

**Results:**

The NMR spectra analysis identified quantitative differences for sixteen metabolites out of forty, including branched chain and aromatic amino acids, glucose, and lipids, thus revealing interesting possible associations with the phenotypes showed by vitamin B6 deficient flies.

**Conclusions:**

Our results validate *Drosophila* as a suitable model to study in depth the molecular mechanisms underlying human diseases associated with vitamin B6 deficiency and confirmed that 4DP treatment is effective in inducing vitamin B6 deficiency.

**Supplementary Information:**

The online version contains supplementary material available at 10.1007/s11306-025-02236-0.

## Introduction

Pyridoxal 5’-phosphate (PLP) is the biologically active form of vitamin B6, involved as a cofactor in 4% of cellular metabolic reactions, primarily amino acid degradation and synthesis, and one-carbon unit metabolism. PLP binds at the active site of the enzymes as a Schiff base with a lysine residue and catalyses transamination, racemization, decarboxylation, elimination and synthesis reactions involving amino-substrates (John, 1995). In addition to the cofactor role, PLP is an antioxidant molecule able to counteract oxygen reactive species (ROS) and advanced glycation end products (AGEs) (Mascolo & Vernì, 2020). In humans and animals, PLP is synthesized from dietary B6 vitamers in the salvage pathway where the concerted action of pyridoxal kinase (PDXK) and pyridoxine/pyridoxamine phosphate oxidase (PNPO) produces PLP from pyridoxal (PL), pyridoxine (PN) and pyridoxamine (PM) (di Salvo et al., 2011). Vitamin B6 is naturally abundant in many foods (Contestabile et al., 2020), making dietary deficiency of this micronutrient rare in developed countries. However, secondary deficiency may result from various conditions, including genetic factors, drugs such as isoniazid, cycloserine and penicillamine (Lainé-Cessac et al., 1997), kidney diseases and malabsorption syndromes including celiac disease and inflammatory bowel diseases (Merrill & Henderson, 1987). In addition, PLP concentrations are low in older people, in people with alcohol dependence (Cravo & Camilo, 2000), in pregnant women, obese individuals and diabetic patients (Ferro et al., 2017; Merrill & Henderson, 1987; Mascolo & Vernì, 2020).

Epidemiological and experimental studies indicated an evident inverse association between vitamin B6 levels and diabetes, as well as a clear protective effect of vitamin B6 on diabetic complications (Mascolo & Vernì, 2020). Additionally, vitamin B6 deficiency has been also associated with impaired immune response (Qian et al., 2017) and to cancer risk (Merigliano, 2018). To date, most of the effects due to PLP deficiency are not clearly attributable to specific molecular mechanisms. This is largely due to the limitations of existing studies, which have predominantly been conducted in humans and often faced challenges such as flawed study designs and imprecise dietary assessments. Thus, studies in animal models can help address these problems, and give the possibility of investigating underlying mechanisms.

The consequences of vitamin B6 deficiency have been studied in *Drosophila melanogaster* and the results obtained helped to shed light on molecular mechanisms and pathways behind the role played by vitamin B6 in some human diseases. In *Drosophila* it has been demonstrated for the first time that mutations in *dPdxk* or *sgll*^*PNPO*^ genes result in chromosome aberrations (CABs) strongly enhanced by sucrose, glucose or fructose treatments (Marzio et al., 2014) and also that low PLP levels produce diabetic phenotypes due to insulin resistance (IR) (Marzio et al., 2014). Intriguingly, diabetic flies depleted of PLP displayed a huge genomic damage than healthy flies, thus suggesting that in diabetic people low PLP levels may increase the risk of cancer by promoting genome instability (Merigliano et al., 2018). Although how IR is established is yet an open question, studies in flies contributed to clarify the complex relationship linking the vitamin B6 to diabetes indicating that decreased PLP levels, found in diabetic people, can be both cause and consequence of the disease (Mascolo & Vernì, 2020). Additionally, flies carrying mutations in *sgll*^*PNPO*^ gene displayed neurological defects (Chi et al., 2019) accordingly with the finding that human *PNPO* gene has been linked to a neonatal epilepsy form (Mills et al., 2005). Further studies in *Drosophila* suggested a molecular basis for the inverse relationship linking vitamin B6 to some cancer types. It has been demonstrated that PLP deficiency promotes cancer onset and transformation by both producing genome instability and impairing the synthesis of thymidylate (dTMP) regulated by the PLP dependent enzyme serine hydroxymethyltransferase (Gnocchini et al., 2022; Pilesi et al., 2024).

The effects of vitamin B6 deficiency have been studied in bacteria, fungi, plants and animals using analogs, i.e. compounds that are structurally similar to B6 vitamers but lack the biochemical properties for enzyme catalysis. Among them, the most used compound was 4-deoxypyridoxine (4DP) and its phosphorylated derivative 4-deoxypyridoxine 5’-phosphate (4DPNP). Once entered the cell, 4DP is phosphorylated by pyridoxal kinase into 4DPNP, which is thought to act as a competitor of PLP (Coburn, 2017). By mimicking the structure of PLP, 4DPNP competes for binding at the active sites of PLP-dependent enzymes, reducing their activity and thereby impacting the downstream metabolic processes. 4DP and 4DPNP also act as inhibitors of enzymes involved in vitamin B6 metabolism. It has been demonstrated that 4DP competitively inhibits the in vitro activity of human PDXK (Hanna et al., 1997; Kästner et al., 2007) and that 4DPNP inhibits PNPO (Salamon et al., 2009). In this case the effect is that of inhibiting PLP synthesis and as a consequence to reduce the fraction of enzymatically active PLP-dependent enzymes. Inhibition of PLP-dependent enzymes and vitamin B6 metabolism by 4DP has been also demonstrated in bacteria (Vu & Downs, 2022; Babor et al., 2023). Moreover, 4DP also competitively inhibits the uptake of extracellular B6. Alterations in B6 transport have been noted in various mammalian tissues (Coburn, 2017) and in *Escherichia coli* (Babor et al., 2023).

4DP has been also used on *Drosophila*, in which effects on growth and morphology have been observed (Coburn, 2017). Given the similarities in the mechanism of action of 4DP observed in humans and bacteria, it is reasonable to assume that the effects of 4DP observed in *Drosophila*, which are similar to those observed in flies carrying mutations in genes involved in PLP biosynthesis, are due to the same mechanism, especially since these effects are rescued by the administration of PLP (Marzio et al., 2014; Merigliano et al., 2018; Gnocchini et al., 2022; Pilesi et al., 2024).

Nowadays, metabolomics undoubtedly represents one of the most effective approaches capable to guide in deeper understanding of metabolic responses to pathophysiological stimuli, genetic modifications or the characterization of phenotypes associated with specific diseases (Nagana Gowda & Raftery, 2021; Huang et al., 2022; Moco, 2022). Regarding the analytical platforms, nuclear magnetic resonance spectroscopy (NMR) presents many advantages being a non-destructive, high reproducible technique and allowing in a single experiment the simultaneous quali-quantitative analysis of a biological matrix without any complex sample pretreatment.

Therefore, to better validate *D. melanogaster* as a suitable model organism to study the role of vitamin B6 in human diseases, in this study we performed an NMR-based metabolomic analysis, by characterizing the changes of the metabolome induced by 4DP on *D. melanogaster* third instar larvae. Altered metabolites found in this study may help identify the mechanisms through which vitamin B6 deficiency affects some human diseases.

## Materials and methods

### Growth medium and cytological analysis

Flies were maintained at 25 °C on a standard medium containing in 100 mL: 0.68 g agar, 6.52 g yeast, 3 g flour, 600µL propionic acid, and 5.13 g sucrose. 4-deoxypyridoxine (4DP, Sigma Cat. No. D0501) was dissolved into the standard medium at 2 mM final concentrations according to (Pilesi et al., 2024). *Oregon-R* was used as wild type stock.

4DP treatment delays the larval development. Thus, we analyzed and compared untreated and 4DP-treated larvae after they exited the food, to be sure that they reached the third stage. This happened 8 days post eclosion in untreated wild-type larvae and 11 days post eclosion in 4DP-treated wild type larvae.

Chromosome cytology was performed on larval brains dissected from wild-type untreated or 4DP-treated larvae according as described in (Merigliano et al., 2018). Observations were carried out using a Zeiss Axioplan fluorescence microscope equipped with CCD camera (Photometrics CoolSnap HQ). Statistical analysis was performed using the unpaired two tailed t-test. *P* < 0.05 was considered significant.

### Blue-dye feeding assay

Five groups of eight third instar larvae (four males and four females) were transferred onto standard or supplemented with 2mM 4DP fresh food medium containing 4% (w/v) blue food dye (Blue Dye no. 1, Merck). After feeding larvae were washed and homogenized in 100 µl of 1X PBS. The homogenate was centrifuged at 35,000 rpm for 10 min and absorbance for blue dye at 629 nm was read using spectrophotometer (Multiskan GO version 1.00.40 Thermo scientific). This experiment was replicated to quantify the amount of ingested food by measuring the ratio between the blue area/ total body area of larvae (using the image J software) on pictures acquired with the Axiocam 208 color camera (Zeiss) connected to the Stemi 305 stereomicroscope.

### Measurements of B6 vitamers by HPLC

Samples of 50 frozen larvae were resuspended in 300 µl 50 mM KP_i_, pH 7.5, homogenized with a small pestle and sonicated. An aliquot of supernatant was used to determine the total protein concentration with Bradford assay, whereas the rest was treated with 4 M KOH and 25% HClO_4_. The mixture was centrifuged, and an aliquot analysed by HPLC. The separation of vitamers was performed using an HPLC system (Vanquish Core, ThermoFisher Scientific), equipped with both an UV (290 and 340 nm) and a fluorescence detector (excitation 290 nm, emission 395 nm), and using a C18 Acclaim column (4.6 × 150 mm, particle size 5 μm, ThermoFisher Scientific) with 33 mM phosphoric acid and 8 mM 1-octanesulfonic acid, adjusted to pH 2.2 with KOH, as mobile phase A, and 80% acetonitrile (vol/vol), as mobile phase B. The linear gradient was from 1% mobile phase B to 2% B for 5 min, 2% B to 20% B for 10 min, 20% B to 30% B for 5 min, and 30% B to 90% B for 5 min. For quantification of vitamers, 10 µM pure PL, PMP, PLP PNP or 4DP, treated as the samples, were used as standard.

### Sample preparation for NMR analysis

For each category, five samples each containing 20 (twenty) animals were analyzed by NMR spectroscopy. All samples were extracted following a modified Bligh-Dyer protocol as reported in (Giampaoli et al., 2023). Frozen larvae (−80 °C) were transferred in cylindrical centrifuge tubes and added with 2mL of cold methanol (4 °C) to ensure metabolic quenching. Each sample was then homogenized with a small pestle and added with chloroform (2 mL) and deionized water (1 mL). After an overnight storage at 4 °C, the samples were centrifugated at 10,000 rpm for 20 min and then the two phases carefully separated and evaporated under a nitrogen flux. The dried phases were finally stored at −80 °C until NMR analysis. The hydrosoluble phase was resuspended in D_2_O containing 3-(trimethylsilyl)-propionic-2,2,3,3-d_4_ (TSP) 2mM as internal chemical shift and concentration standard, while the liposoluble one was resuspended in CDCl_3_ containing hexamethyldisiloxane (HMDSO) 2mM as concentration reference. All solvents and standards were purchased from Sigma-Aldrich (St. Louis, MO, USA).

### NMR experiments

All spectra were collected in random order at 298 K on a JEOL JNM-ECZR (JEOL Ltd., Tokyo, Japan) spectrometer equipped with a magnet that works at 14.09 T and at 600.17 MHz for the ^1^H frequency using such acquisition parameters: a spectral width of 9.03 kHz (15ppm), a relaxation delay of 7.72s, 64k points and 128 scans. For aqueous samples a presaturation pulse sequence (*presat*) was employed for solvent signal suppression. Assignment of resonances was achieved conducing bidimensional experiments ^1^H-^1^H TOCSY at 298 K with a spectral width of 15 ppm in both dimensions, using 8k x 256 data points and 80 ms as mixing time; and ^1^-^13^ C HSQC setting as spectral widths 9.03 kHz (15 ppm) for the proton dimension and 30 kHz (200 ppm) for the carbon dimension, with 8k × 256 data points for two dimensions and 2 s of repetition delay. Furthermore, confirmation for metabolites identification coming from bibliography comparison, Chenomx NMR Suite 9.0, and Human Metabolome Database (HMDB) (Wishart et al., 2022), as also reported elsewhere (Gogna et al., 2015). One-dimensional NMR spectra have been manually phased, baseline corrected, and chemical shift referred (using CHCl3 or TSP signal for organic or aqueous respectively) using ACD Lab 1D-NMR Manager Ver. 12.0 software (Advanced Chemistry Development, Inc., Toronto, ON, Canada). JEOL Delta v5.3.1 software (JEOL Ltd., Tokyo, Japan) was instead used for two-dimensional experiments. TSP or HMDSO have been used as quantitative internal standards, according to the general formula:

$$\:{C}_{m}=\:\frac{{A}_{m}}{{A}_{IS}}\times\:\frac{{H}_{IS}}{{H}_{m}}\times\:{C}_{IS}$$

where C_m_ is the concentration of the metabolite, A_m_ is the area of the metabolite signal, H_m_ is the number of protons generating the metabolite signal, C_IS_ is Internal Standard’s (IS) concentration (TSP or HMDSO respectively for aqueous or organic fraction), A_IS_ is the area of IS signal and H_IS_ is the number of proton generating IS signal. Due to the overcrowding of ^1^H NMR spectra, only those signals that did not overlap with other resonances were considered for integration. All metabolites concentration were finally expressed as µmol/g of fresh weight.

### Statistical analysis for metabolomics data

Given the high correlation of variables resulting from a metabolomic analysis, the data obtained were first considered from a multivariate point of view. To evaluate any samples spontaneous grouping, unsupervised PCA were applied to the entire dataset after mean-centering and scaling. Besides, to highlight the role of each metabolite in distinguish between the groups a PLS-DA model has been built, using as validation procedure the double cross validation method (Szymańska et al., 2012). Accuracy, Precision, Sensitivity and Specificity were then employed as validation parameters and significance of such indices was assessed by 1000 random permutation of samples labels (Figure S2). Significant variables for regression have been selected taking into account both Regression Coefficients and the Variable Importance in Projection (VIP) criterion, only variables whose Regression Coefficient sign remained consistent during cross validation procedures and whose VIP value is greater than 1 were considered significant for the model (C & Chi-Hyuck J, 2005).

For univariate analysis, data were primarily tested for normality and homoscedasticity of distributions between classes using Shapiro-Wilk and Brown-Forsythe tests; then paired-samples t-test or Wilcoxon test were applied accordingly. All tests were two-sided, with statistical significance set at *p* < 0.05. All analyses were performed using in-house routines running under MATLAB environment (The MathWorks, Natick, MA, USA).

## Results

In this work we carried out an NMR based metabolomic study to evaluate changes in metabolism induced by 4DP feeding in order to confirm the feasibility of *Drosophila* as a suitable model for human vitamin B6-related diseases, as well as to identify possible molecular mechanisms at the basis of phenotypes observed in flies depleted of vitamin B6 (Marzio et al., 2014; Mascolo et al., 2020; Pilesi et al., 2024).

### 4DP feeding decreases PLP content

**Fig. 1:**
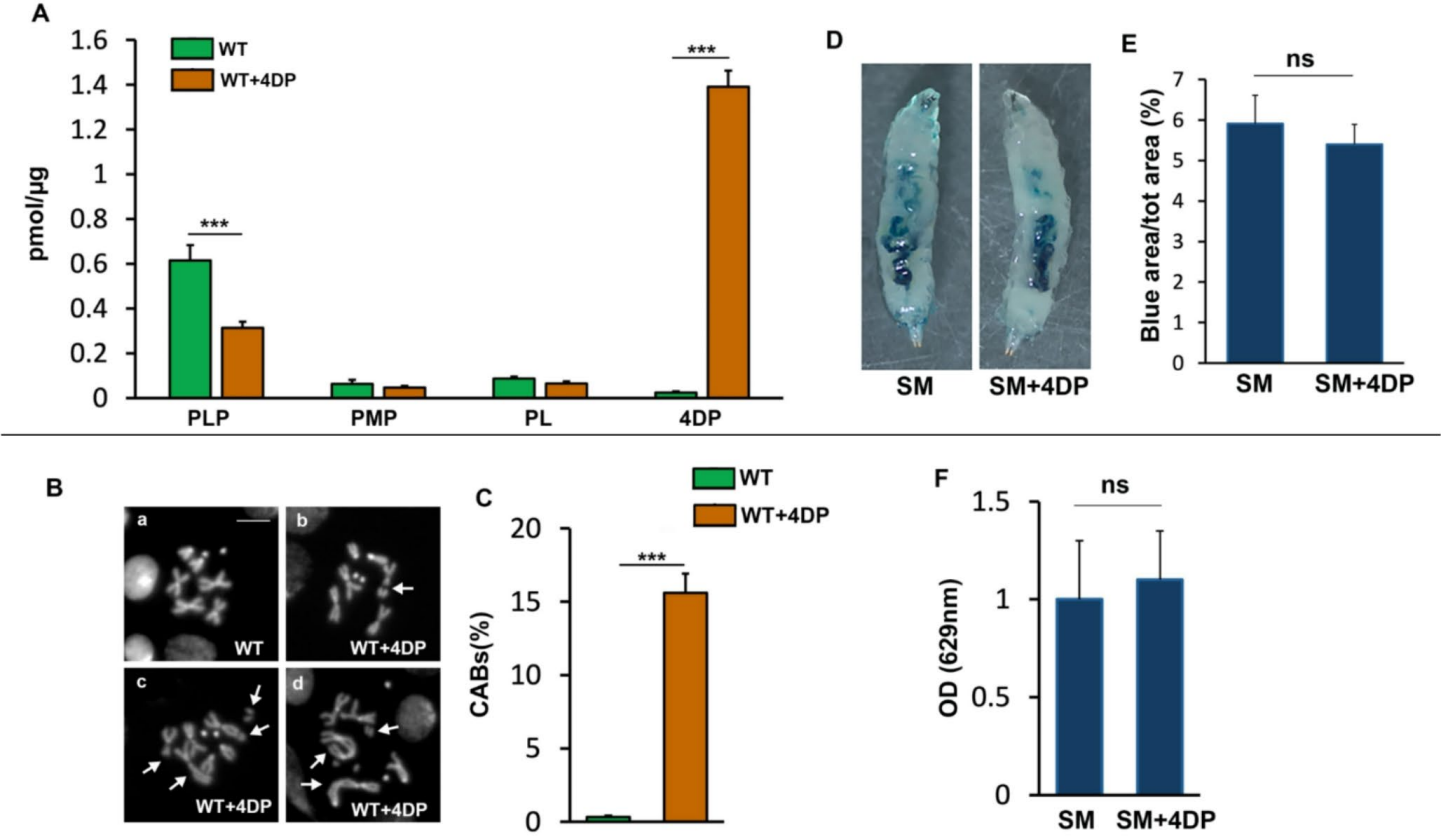
4DP feeding induces vitamin B6 deficiency in *Drosophila* wild type larvae. **A** 4DP treatment reduces PLP levels in larval extracts as revealed by HPLC analysis. Error bars, SEM ***P < 0.001, (unpaired t-test). Vitamers were measured in biological triplicates as explained in Materials and methods and normalized on the basis of the amount of total cellular proteins. **B** Examples of chromosome aberrations (CABs) in neuroblasts from wild type larvae untreated or treated with 4DP. (**a**) Normal female metaphase; (**b**) isochromatid deletion of a major autosome; (c)(d) metaphases with aberrations involving multiple chromosomes (arrows); Scale bar, 5 μm. 4DP = 4-deoxypyridoxine. WT = wild type Oregon R strain **C** Quantification of results. Error bars, SEM. ***P < 0.001 (unpaired t-test). Total number of examined cells in at least three independent experiments: WT 4DP *n* = 881 (5 brains); WT *n* = 1303 (8 brains). **D** 4DP did not increase the feeding rate in wild type larvae. The ingested blue food is evident in the intestinal tract of larvae. SM = standard medium **E** Quantification. Error bars, SEM. ns = not significant (unpaired *t*-test). *n* = 40 **F** OD measurement. Error bars, SEM. ns = not significant (unpaired *t*-test). *n* = 40

Before proceeding to NMR-analysis, we measured vitamin B6 vitamers in extracts from wild-type untreated or 4DP-treated larvae. Extracts from treated larvae, analysed by HPLC, contained high levels of 4DP and showed a statistically significant halving of PLP levels compared to untreated larvae. This corresponds to a decrease, although not statistically significant, in PL and PMP levels. The amount of PNP was not detected (Fig. 1A).

Morever, we also confirmed that 4DP-fed larvae displayed chromosome aberrations (CABs) in larval brains (15% vs. 0.5% in controls) as previously described (Merigliano et al., 2018) (Fig. 1B, C).

### Blue dye assay

To be sure that possible metabolic alterations were not due to an increased feeding rate, we performed a feeding assay in which wild type larvae were placed for 3 h on a standard food with addition or not of 4DP and containing 4% of Blu dye n.1 (Blue brilliant FCF). As reported in Fig. 1 the presence of 4DP did not increase the feeding rate of the larvae as estimated by calculating the percentage of ingested dye (Fig. 1D, E) and by spectrophotometric measurements (Fig. 1F).

### NMR-based metabolomics

Spectra of WT larvae treated or not treated with 4DP were compared and no qualitative differences between the two categories were found. Representative ^1^H NMR spectra for both fractions with molecules assignment are shown in Fig. S1. Forty metabolites belonging to different chemical classes such as amino acids, organic acids, amines, fatty acids and lipids were univocally identified from both the hydroalcoholic and the chloroform extracts based on signal chemical shift, multiplicity, TOCSY and HSQC and HMBC. A complete list of all metabolites with the relative resonance chemical shifts is also reported in Table S1. Furthermore, chosen a single signal, all identified molecules have been quantified.

Quantities were finally expressed in µmol/g normalizing each concentration to the sample fresh weight and reported in Table S2. The PCA carried out on the whole data matrix provided a model whose first three components explained approximately 80% of the total variance of dataset, with the first component (PC1) accounting for 50,5% and the second one (PC2) for 19%.

**Fig. 2:**
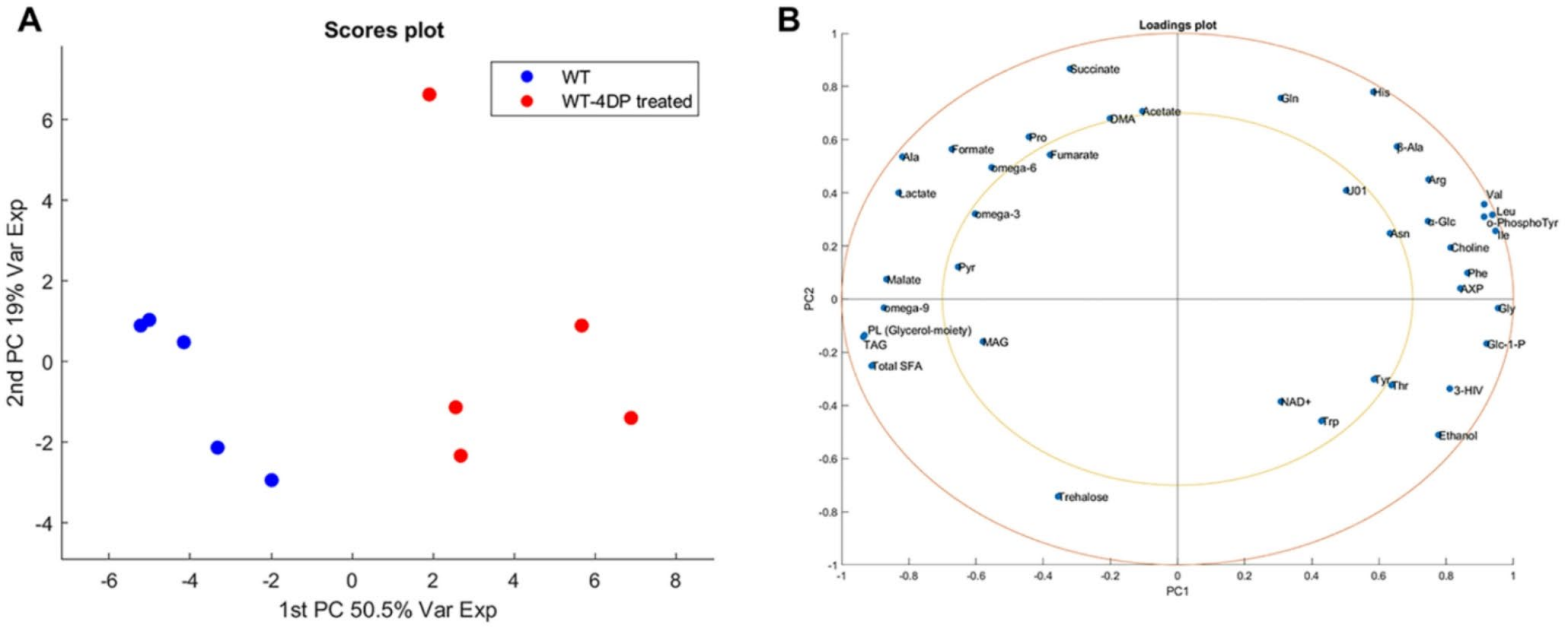
Unsupervised multivariate statistical analysis. **A** Scores plot of PCA performed on WT larvae (blue) and WT-4DP treated larvae (red). **B** loadings plot

From the scores plot (Fig. 2A) a spontaneous grouping, according to treatment, was identified along the PC1 which thus appears to capture the entire variance related to this factor. WT larvae showed negative values of PC1, while extracts from larvae treated with 4DP (WT-4DP treated) showed positive values of the latter. The role of each metabolite in discriminate two groups can be then appreciated from the PCA loadings plot (Fig. 2B) where a good separation, comparable to samples’ scores, was noted. In this case all variables with positive values on PC1 contribute to distinguish treated group from controls.

**Fig. 3:**
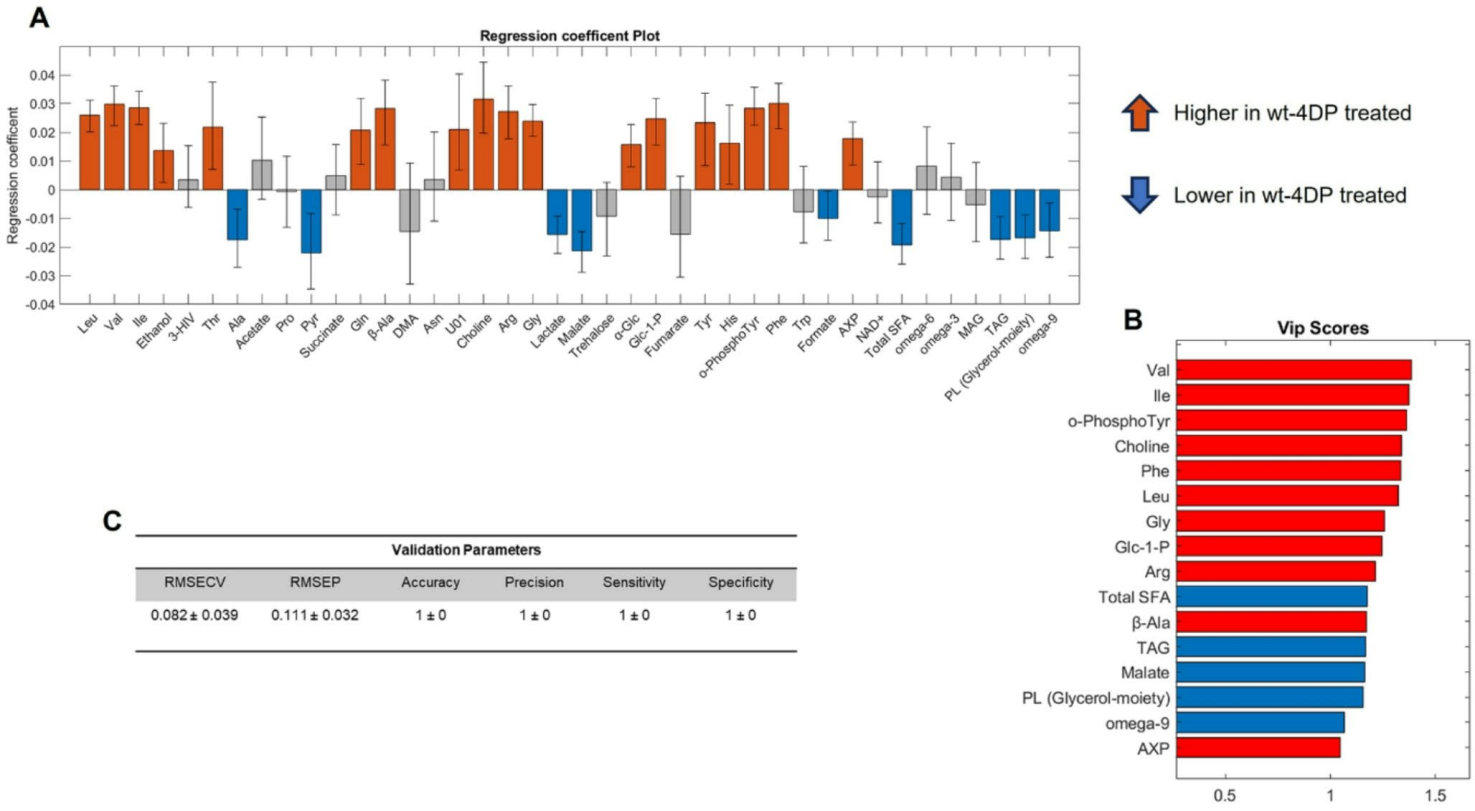
Supervised multivariate statistical analysis **A** Regression Coefficients for PLS-DA model. **B** Vip Scores. **C** Model validation parameters. RMSECV stands for Root Mean Square Error during Cross Validation procedure, RMSEP stands for Root Mean Square Error in Prediction

Besides, with the aim of selecting only those variables that majorly contribute to the discrimination, a supervised PLS-DA model was built (Fig. 3A, B). The model, realized with two latent variables (LVs) that account together for 73.45% and 99.27% of total variance along X and Y respectively, proved to be very robust with the validation parameters reported in Fig. 3C.

According to the above-mentioned criteria sixteen variables were considered as significant. Valine (Val), Isoleucine (Ile), Leucine (Leu), Phenylalanine (Phe), Ortho-Phospho-Tyrosine (O-Phospho-Tyr), Arginine (Arg), Glycine (Gly), beta-Alanine (beta-Ala), Choline, Glucose-1-Phosphate (Glc-1-P), Adenosine-X-Phosphate (AXP), have shown a positive covariation with 4DP treatment. Malate, Total Saturated Fatty Acid (Total SFA), Triacylglycerols (TAG), Phospholipids (PL Glycerol-moiety) and Omega-9 have been indeed found negatively correlated with treatment.

**Fig. 4:**
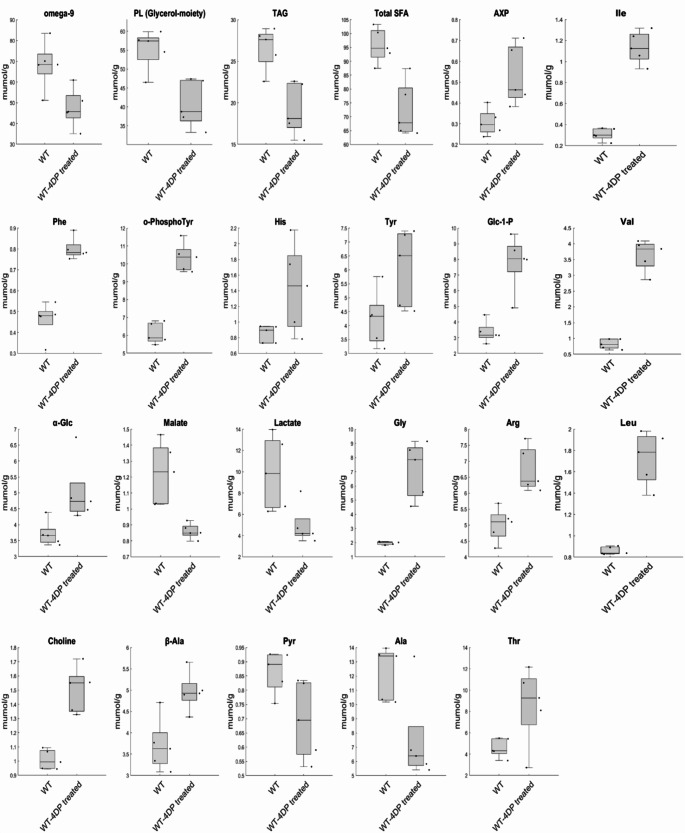
In figure are reported as boxplot only those metabolites considered significant with a confidence level of 95% at the univariate statistical analysis. Details are reported in “Statistical analysis for metabolomics data” section

At the same time, from a univariate point of view, larvae treated with 4DP were characterized from higher levels of Leu, Val, Ile, Threonine (Thr), beta-Ala, Choline, Arg, Gly, alpha-Glc, Glc-1-Phosphate, Tyrosine (Tyr), Histidine (His), O-Phospho-Tyr, Phe and AXP. Instead, metabolites as Ala, Pyruvate (Pyr), Lactate, Malate, Total SFA, TAG, Phospholipids and omega-9 have been found in lower concentrations in treated larvae than in untreated larvae, as reported in Fig. 4.

## Discussion

Vitamin B6 deficiency has previously been studied in flies carrying mutations in genes involved in PLP biosynthesis, such as *Pdxk* and *Sgll*^*/PNPO*^, and resulted in genome instability, diabetes, impaired lipid metabolism and altered nucleotide pool, consistently with the role of PLP as an antioxidant, as well as with role of this coenzyme implicated in a plethora of metabolic activities (Marzio et al., 2014; Mascolo et al., 2020). Similar phenotypes were also produced by PLP antagonists, such as 4DP and ginkgotoxin, that were also found to impact on cancer growth and invasiveness (Pilesi et al., 2024). However, the effects of PLP deficiency have never been examined at a metabolic level in flies. Thus, the purpose of our work has been to identify possible metabolic biomarkers associated to PLP deficiency that could help to identify the pathways involved in specific PLP-associated diseases.

Metabolomics has been successfully applied in *Drosophila* often revealing new insights into gene function and metabolism, difficult to obtain using other approaches (Cox et al., 2017). For example, NMR was applied in studies concerning the circadian regulation of metabolites and in works that analyzed the response of metabolome to heat stress (Cox et al., 2017). Additionally, this approach was successfully employed to assess the feasibility of *D. melanogaster* as a model for Huntington disease (Singh et al., 2017).

**Fig. 5:**
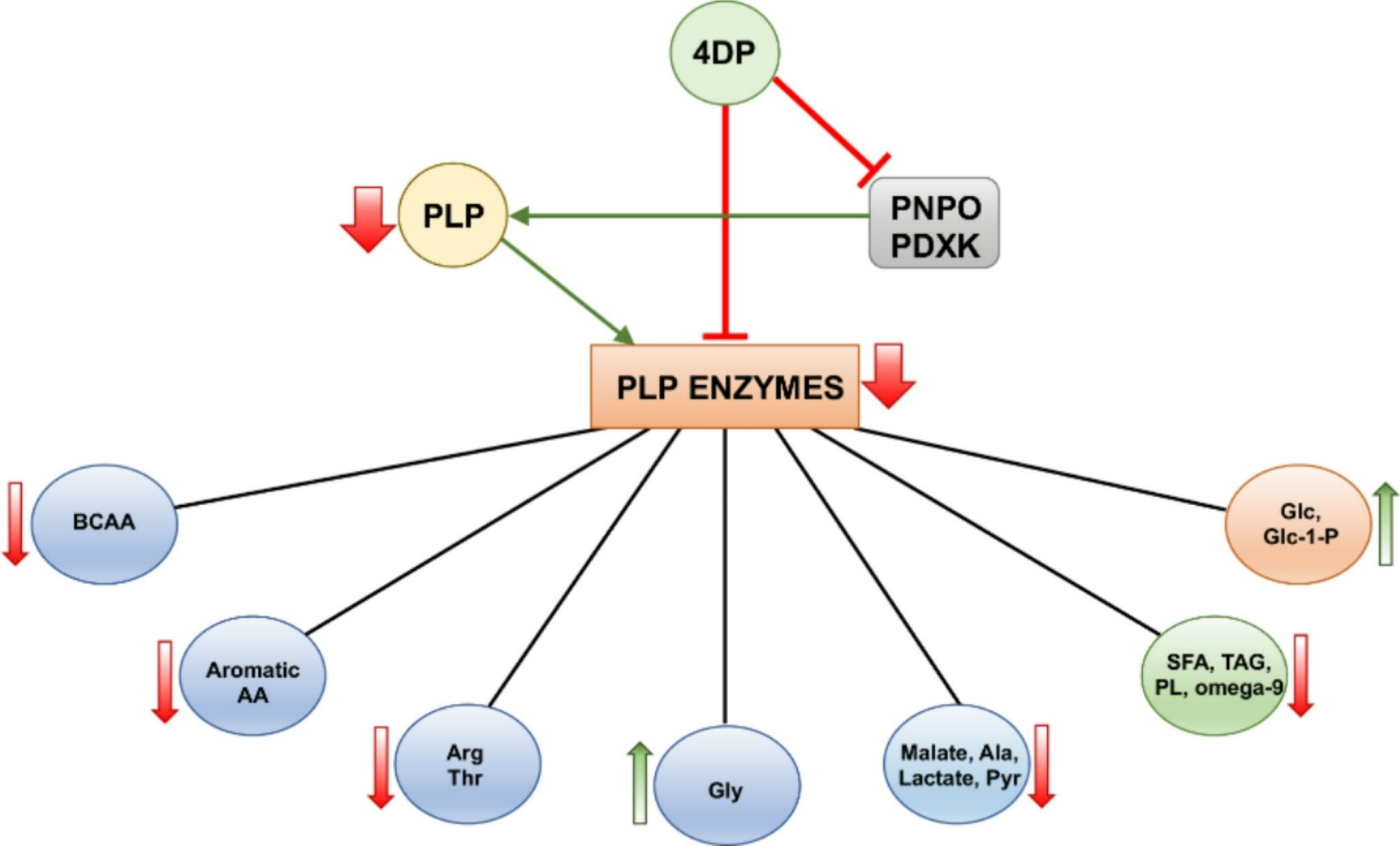
Scheme illustrating the action mechanism of 4DP. By mimicking the structure of pyridoxal phosphate, 4DP competes for binding at the active sites of PLP-dependent enzymes, reducing their activity and thereby impacting the downstream metabolic processes. Additionally, it inhibits the enzymes involved in vitamin B6 metabolism, thus inhibiting PLP synthesis and hence reducing the fraction of enzymatically active PLP-dependent enzymes

In our model organism, *Drosophila*, 4DP, obtained from food, is expected to inhibit the PLP synthesis enzymes (PNPO and PDXK) (Coburn, 2017), leading to a decrease in PLP levels (Fig. 5).

In humans, the concentration of plasmatic PLP is about 50 nm/L and values of 30 nm/L and 20 nm/L are considered as a marginal and severe deficiency, respectively (Leklem, 1990). Here we found that 4DP feeding halved the PLP concentration in the larvae, thus inducing, by analogy, a marginal to severe deficiency which confirmed the validity of our model. The presence of 4DP, combined with reduced PLP levels, leads to a decrease in the enzymatically active PLP-dependent enzymes (Fig. 5). This phenomenon may explain the significant quantitative differences observed in several metabolites in 4DP-fed larvae, as revealed by our analysis. In fact, due to its chemical properties, PLP facilitates enzymatic reactions involved in catabolism and interconversion of most amino acids (Liang et al., 2019). Consistent with this, in this study we found higher concentrations of branched chain amino acids (BCAAs: Val, Ile, Leu), aromatic amino acids (Phe, Tyr, O-PhosphoTyr, His), Arg and Thr in extracts from larvae reared on 4DP medium, thus suggesting an impaired activity of PLP-dependent enzymes such as decarboxylases and aminotransferases. In particular, Branched-chain amino acid aminotransferase (BCAT) catalyzes the conversion of BCAAs and α-ketoglutarate into branched chain α-keto acids and glutamate (Nong et al., 2022). Differently from humans that possess two BCAT isoforms, cytosolic BCAT1 and mitochondrial BCAT2 (Nong et al., 2022), the fly genome harbors only one BCAT gene functionally equivalent to BCAT2 (Martelli et al., 2024). It is therefore expected that in flies a reduced activity of this enzyme -due to reduced PLP availability- can lead to BCAA accumulation.

Growing evidence indicates that Leu, Ile, and Val have a prominent role in the activation of the Target of Rapamycin (TOR) pathway (Hara et al., 1998) and elevated circulating BCAA levels have been associated with insulin resistance, diabetes and obesity. In contrast, enhancing BCAA catabolism improves glucose homeostasis in metabolic disorders, such as obesity and Type 2 diabetes (Vanweert et al., 2022).

We previously demonstrated in *Drosophila* that PLP deficiency caused by *Pdxk* or *sgll*^*/PNPO*^ mutations, as well as by 4DP feeding, increased the hemolymph glucose content due to insulin resistance established with mechanisms not yet clarified (Marzio et al., 2014; Mascolo et al., 2020; Merigliano et al., 2018). In line with these data, the metabolomic analysis of 4DP-treated larvae here confirmed that PLP deficiency increases the glucose levels (Fig. 4). Thus, we could speculate that insulin resistance associated with vitamin B6 deficiency may, in part, be related to elevated levels of BCAAs. Moreover, our data suggest that studies in flies may contribute to shed light on the molecular mechanisms and pathways linking BCAAs to diabetes.

The increased levels of aromatic amino acids, such as tyrosine and phenylalanine, that we found in larvae treated with 4DP were also observed in rats by Stanley et al. (1985) who described a decrease in catabolism of Tyr and Phe in isolated liver cells of animals subjected to pyridoxine-free diet, caused by reduced activity of the enzyme tyrosine aminotransferase. Elevated levels of aromatic amino acids may indicate reduced function of aromatic amino acid transaminase or downstream enzymes like phenylalanine hydroxylase (Peñalva, 2001).

Additionally, in line with our data, the accumulation of both BCAAs and aromatic amino acids has been also found in culture cells in which PLP levels were decreased by the depletion of the PLPHP protein involved in the control of PLP homeostasis (Ciapaite et al., 2023).

The observed increase in Gly concentration could be explained by the fact that both glycine decarboxylation through the glycine cleavage system (GCS) and the reversible glycine-serine interconversion catalyzed by the serine hydroxymethyltransferase (SHMT) enzyme depend on PLP as a coenzyme. Accordingly, an increment in serum and urine of glycine concentrations was also found in healthy volunteers with marginal vitamin B6 deficiency (Lamers et al., 2009). However, it must be pointed out that the changes occurring in glycine content are strongly organ-dependent in mammals. In greater details, glycine is lower in brain (Ramos et al., 2017), while it is more abundant in blood, liver and muscle (Runyan & Gershoff, 1969).

At a systemic level we found a decrease in Krebs cycle intermediates (Malate) and related molecules (Lactate, Alanine, Pyruvate). Much of the alanine is derived from BCAAs via the action of PLP-dependent transaminases (Holeček, 2018; Haymond & Miles, 1982). While malate itself is not directly synthesized via PLP-dependent reactions, the precursor amino acids feeding into the TCA cycle, such as glutamate (via α-ketoglutarate) and aspartate (via oxaloacetate), rely on PLP-dependent transaminases. Concerning the observed decrease in lactate and pyruvate, it should be considered that PLP is the coenzyme for alanine transaminase, which catalyzes the reversible transamination between alanine and pyruvate. A reduction in pyruvate availability leads to a decrease lactate production via lactate dehydrogenase. Therefore, PLP deficiency disrupts metabolic flux, leading to diminished concentrations of these key metabolites. This finding is partially in line with what has been observed on mammalian cardiac muscle tissues (Kumrungsee et al., 2019) and on PLPHP-deficient cells which displayed a depletion of TCA cycle intermediates downstream of isocitrate dehydrogenase 1 and 3, possibly due to decreased anaplerosis from amino acids (Ciapaite et al., 2023).

4DP-treated larvae also displayed a generalized lower amount of lipids (Total SFA, TAG, Phospholipids, and omega-9). Low PLP levels disrupt several metabolic pathways that are critical for lipid metabolism, including the synthesis of acetyl-CoA (from TCA cycle intermediates, such as oxaloacetate and α-ketoglutarate, which generate citrate), glycerol-3-phosphate (deriving from pyruvate), and amino acid-derived precursors, like serine. This leads to a reduction in total SFAs, TAG, phospholipids, and omega-9 fatty acids. Although it is known that vitamin B6 deficiency affects lipid metabolism, the underlying mechanisms remain poorly understood and require further studies. The actual knowledge indicates that vitamin B6 deficiency reduces plasma (n-3) and (n-6) polyunsaturated fatty acid concentrations, possibly due to reduction in the rate of desaturation processes (Zhao et al., 2013). Additionally, it has been proposed that PLP depletion may reduce the adipogenesis by regulating transcription and/or methylation of the adipogenes (Moreno-Navarrete et al., 2016). In line with this last role, in *Drosophila* the depletion of Sgll^/PNPO^ increased the ectopic accumulation of TAGs in the form of droplets in the fat body (the fly liver), consistently with the insulin resistance displayed by these flies (Mascolo et al., 2020). Although our NMR analysis does not reveal the molecular mechanisms underlying the reduction in lipid content in vitamin B6 deficient larvae, it confirmed that low PLP levels have a significant impact on lipid metabolism.

Regarding the carbohydrate metabolism, together with the glucose increase, our analysis revealed a significant increase of Glc-1-P in 4DP-treated larvae. This finding is difficult to explain by considering that PLP is a cofactor of glycogen phosphorylase, which catalyzes the rate-limiting step in glycogenolysis by releasing glucose-1-phosphate. Therefore, it should be expected that the lack of vitamin B6 prevents the demolition of glycogen and decreases the Glc-1-P instead of increasing it. Thereby, we can only hypothesize that the 4DP treatment may induce compensative mechanisms that remodulate carbohydrate metabolism.

Metabolic changes induced by vitamin B6 deficiency have been studied by Gregory et al. (2013) on plasma samples from human subjects fed a low-vitamin B6 diet to induce a marginal deficiency, using an approach which combined mass spectrometry and NMR (Gregory et al., 2013). However, differently from our work, this study reported an increased ratio of glutamine/glutamate and 2-oxoglutarate/glutamate but did not reveal any significant alteration in the profile of 14 other amino acids and 45 acylcarnitines. We can thus hypothesize that the difference with respect to our analysis may be due to the different type of sample examined (plasma vs. whole individuals) and also to the different strategy used to induce vitamin B6 deficiency.

In conclusion, the NMR analysis here applied for the first time in flies to assess the metabolic changes associated with vitamin B6 deficiency revealed marked quantitative alteration of several metabolites in *Drosophila* larvae, mainly attributable to the activity of PLP as coenzyme in reactions affecting the metabolism of amino acids and sugars. However, it should be noted that we have analyzed homogenates of entire individuals. While this approach has been quite successful also in other works (Gogna et al., 2015); Singh et al., 2017), the metabolism of *Drosophila*, like that of any metazoan, is compartmentalized into specific tissues, so this type of analysis could override some quantitative differences expected for a given metabolite in specific tissues. Overall, our data indicate that *Drosophila* is a good model to study molecular mechanisms underlying pathologies associated to vitamin B6 deficiency. In addition, they also indicate that 4DP is a good mean to mimic PLP deficiency in functional studies carried out in flies.

## Supplementary Information

Below is the link to the electronic supplementary material.

Supplementary material 1 (CSV 5.1 kb)

Supplementary material 2 (DOCX 106.7 kb)

## Data Availability

No datasets were generated or analysed during the current study.

## Abbreviations

PLP: Pyridoxal-5’phosphate
PL: Pyridoxal
PMP: Pyridoxamine phosphate
4DP: 4-Deoxypyridoxine
CABs: Chromosome aberrations
Leu: Leucine
Val: Valine
Ile: Isoleucine
3-HIV: 3-Hydroxy-isovalerate
Thr: Threonine
Ala: Alanine
Pro: Proline
Pyr: Pyruvate
Gln: Glutamine
β-Ala: β-Alanine
DMA: Dimethylamine
Asn: Anserine
U01: Unknown compound 1
Arg: Arginine
Gly: Glycine
α-Glc: α-Glucose
Glc-1-P: Glucose-1-phosphate
Tyr: Tyrosine
His: Histidine
O-PhosphoTyr: Phosphotyrosine
Phe: Phenylalanine
Trp: Tryptophan
AXP: Adenosine-X-phosphate
NAD+: Nicotinamide adenine dinucleotide
Tot. SFA: Total amount of saturated fatty acids
Tot. UFA: Total amount of unsaturated fatty acids
MAG: Monoacylglycerols
TAG: Triacylglycerols
PL (Glycerol-mojety): Phospholipids (glycerol mojety)
