# Supplementary material for: Vitamin B6 deficiency produces metabolic alterations in Drosophila
Source: Metabolomics. 2025 Mar 23;21(2):42. doi: 10.1007/s11306-025-02236-0 (PMC11930875; doi:10.1007/s11306-025-02236-0)
Supplement: Supplementary file 2 — Supplementary material 2 (DOCX 106.7 kb) [file 11306_2025_2236_MOESM2_ESM.docx]

**Supplementary Material**
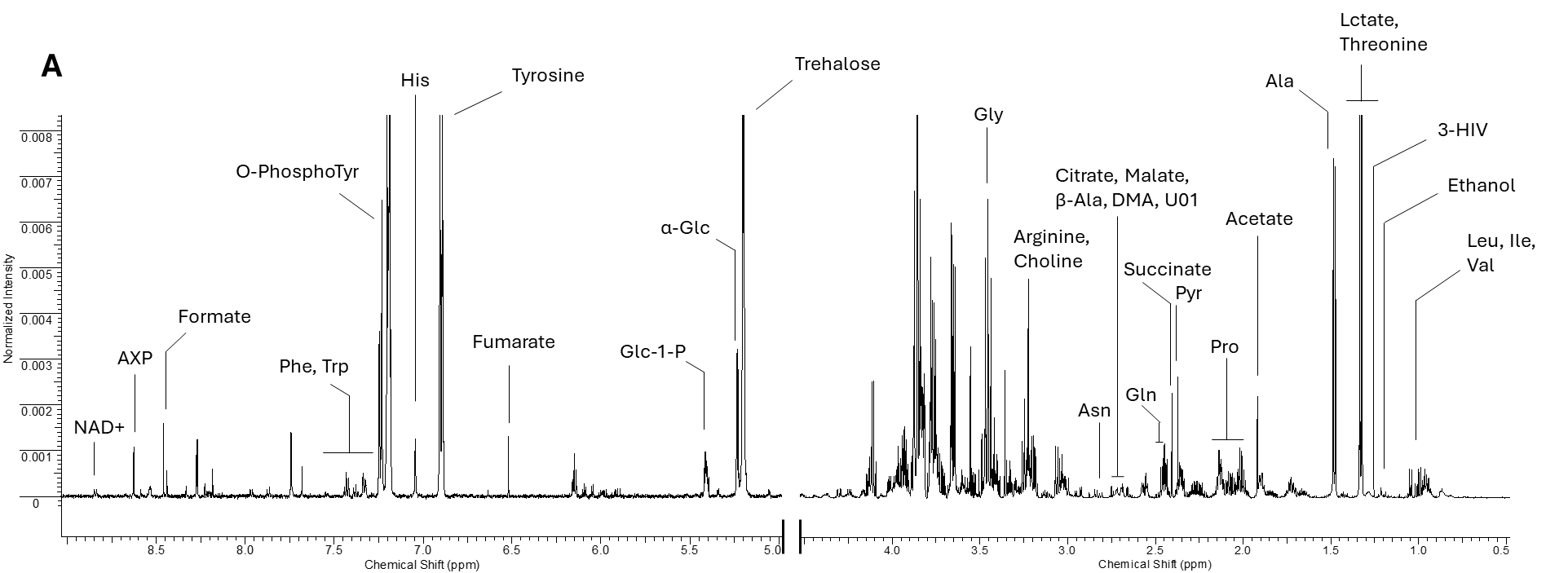


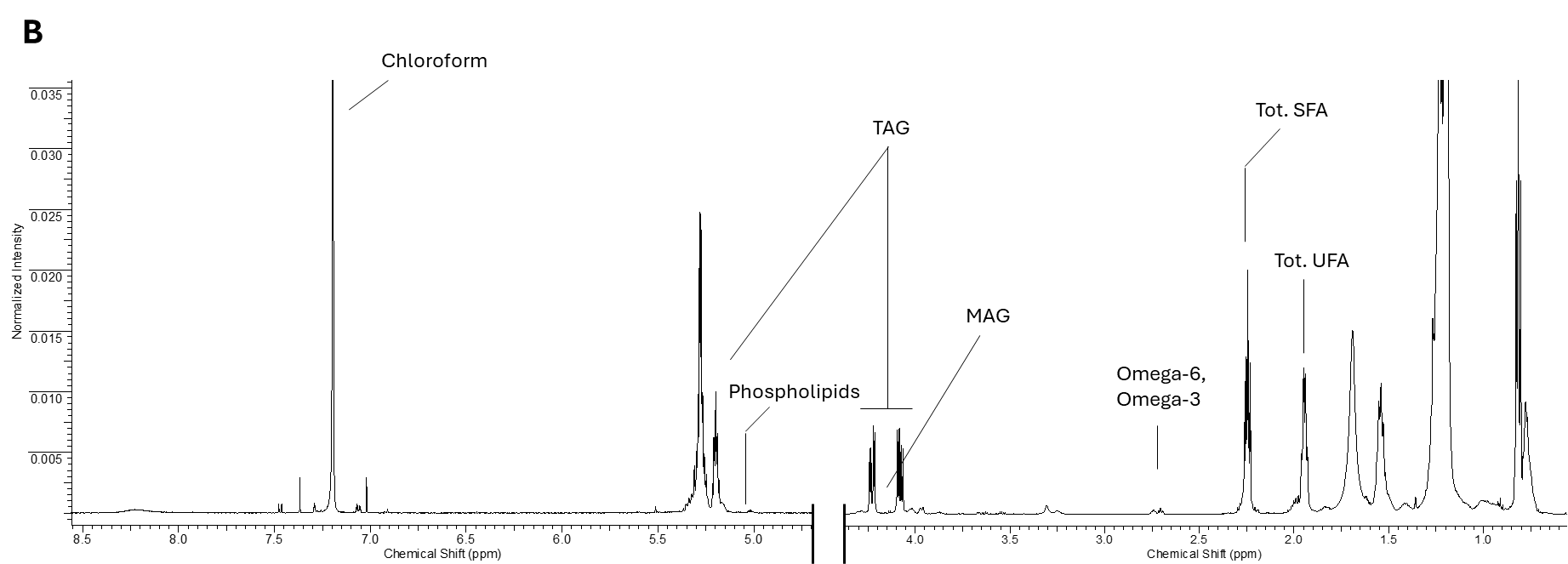


**Figure S1.** Representative spectra of both aqueous (A) and organic (B) fraction.

**Figure S2.** Permutation test performed on three main validation parameters. The continuous lines represent the value of index in the real data, while the histograms show the distribution of each parameter across permutations. On the top are reported the relative p-values for statistical significance.

| Compound | Assignment^a^ | ^1^H (ppm) | Multiplicity^b^ |
| --- | --- | --- | --- |
| Valine (Val) | α -CH  β-CH  **γ-CH_3_**  γ’-CH_3_ | 3,62  2,29  **1,05**  1,00 | m  m  **d**  d |
| Isoleucine (Ile) | α -CH  β-CH  γ-CH  γ'-CH  **δ-CH_3_**  δ’-CH_3_ | 3,69  1,99  1,25  1,49  **1,01**  0,95 | m  m  m  m  **d**  t |
| Leucine (Leu) | α -CH  β-CH_2_  γ-CH_3_  **δ -CH_3_**  **δ’-CH_3_** | 3,74  1,73  1,69  **0,97**  **0,96** | m  m  m  **d**  **d** |
| Ethanol | CH_2_  **CH_3_** | 3,66  **1,19** | q  **t** |
| 3-Hydroxyisovalerate (3-HIV) | **α-CH_2_**  β-CH_3_, β-CH_3_’ | **1.26**  2.35 | **s**  s |
| Alanine (Ala) | α-CH  **β-CH_3_** | 3,77  **1,49** | q  **d** |
| Threonine (Thr) | α-CH  β-CH  **γ-CH_3_** | 3,57  4,24  **1,33** | d  m  **d** |
| Acetate | **CH_3_** | **1,93** | **s** |
| Proline (Pro) | **ϒ-CH2**  β’-CH  β-CH  δ’-CH  δ-CH  α-CH | **2.06**  2.11  2.36  3.36  3.42  4.14 | **m**  m  m  m  m  m |
| Pyruvate (Pyr) | **CH_3_** | **2.46** | **s** |
| Succinate | **α,β-CH_2_** | **2,41** | **s** |
| Glutamine (Gln) | α-CH  β-CH_2_  **γ-CH_2_** | 3,76  2,11  **2,45** | t  dt  **t** |
| β-Alanine (β -Ala) | **CH2**  CH2 | **2.55**  3.17 | **t**  t |
| Dimethylamine (DMA) | **CH3, CH3’** | **2.75** | **s** |
| Asparagine (Asn) | α-CH  β-CH  **β'-CH** | 3,99  2,71  **2,82** | dd  dd  **dd** |
| U01 | - | **2.89** | **s** |
| Choline | **CH3, CH3’, CH3’’** | **3.20** | **s** |
| Arginine (Arg) | α-CH  β-CH_2_  γ-CH_2_  **δ-CH_2_** | 3,76  1,92  1,69  **3,25** | m  m  m  **t** |
| Glycine (Gly) | **α-CH_2_** | **3,69** | **s** |
| Lactate | α-CH  **β-CH** | 4,12  **1,37** | q  **d** |
| Malate | 3-CH_2_  3-CH_2_  **2-CH** | 2.35  2.66  **4.29** | dd  dd  **dd** |
| Trehalose | **CH-1,1’**  CH-2,2’  CH-3,3’  CH-4,4’  CH-5,5’  CH_2_-6,6’ | **5,19**  3,64  3,76  3,44  3,82  3,79 – 3,88 | **d**  m  m  m  m  m |
| α-Glucose | **CH-1**  CH-2  CH-3  CH-4  CH-5  CH_2_-6 | **5,23**  3,55  3,72  3,42  3,84  3,73 – 3,90 | **d**  m  m  m  m  m |
| Glucose-1-Phosphate (Glc-1-P) | **α-CH** | **5.42** | **d** |
| Fumarate | **α,β-CH=CH** | **6,51** | **s** |
| Tyrosine (Tyr) | α-CH  β-CH  β'-CH  C2,6H-ring  **C3,5H-ring** | 3,93  3,15  3,05  7,20  **6,90** | dd  dd  dd  d  **d** |
| Histidine (His) | **C2H ring**  C5H ring | **7.13**  8.02 | **s**  s |
| O-PhosphoTyrosine (O-PhsphoTyr) | α-CH  β-CH  β'-CH  C5H-ring  C3H-ring  C6H-ring  **C2H-ring** | 3.08  3.17  3.98  6.78  6.94  7.13  **7.19** | dd  dd  t  ddd  ddd  ddd  **ddd** |
| Phenylalanine (Phe) | C2,6H-ring  CH-4 ring  **C3,5H-ring** | 7,33  7,39  **7,42** | m  m  **m** |
| Triptophan (Trp) | CH-5 ring  CH-6 ring  CH-7 ring  **CH-4 ring** | 7,20  7,27  7,54  **7,74** | t  t  d  **d** |
| Formate | **CH** | **8,46** | **s** |
| Adenosine Phosphate (AXP) | C2H ring Ade  **C8H ring Ade** | 8,23  **8,58** | s  **s** |
| NAD | **C2H Nam**  C4H Nam  C5H Nam  C6H Nam | **9,34**  8,84  8,29  9,15 | **s**  m  m  m |
| Tot. Fatty Acids | **α-CH_2_** | **2.3** | **m** |
| Tot. Unsatured Fatty Acids | **allilic-CH_2_** | **2.02** | **m** |
| Omega-6 | **homoallilic-CH_2_** | **2.75** | **m** |
| Omega-3 | **homoallilic-CH_2_,CH_2_’** | **2.80** | **m** |
| MAG | **CH (glycerol-mojety)** | **3.60** | **dd** |
| TAG | **CH (glycerol-mojety)** | **4.30** | **dd** |
| Phopholipids | **CH (glycerol-mojety)** | **5.20** | **dd** |

**Table S1.** ^1^H Resonances Assignment

^a^ specific resonance signal used for quantization are reported in bold.

^b^ s: singlet; d: doublet; dd: double doublet; dt: double triplet; t: triplet; q: quartet; m: multiplet
